# Risk factors associated with early death, disease relapse and second primary malignancies in patients with newly diagnosed acute promyelocytic leukemia

**DOI:** 10.3389/fmed.2026.1671705

**Published:** 2026-02-19

**Authors:** Dong-Ming Zhou, Jie He, Min Zhou, Qian Zhang, Lan-Xin Chen, Cui-Ying Yuan, Hui Zeng, Bing Chen

**Affiliations:** Department of Hematology, Nanjing Drum Tower Hospital, Affiliated Hospital of Medical School, Nanjing University, Nanjing, China

**Keywords:** acute promyelocytic leukemia, CNS prophylaxis, early death, relapse, risk stratification, second primary malignancy, treatment era

## Abstract

**Introduction:**

Early death (ED), relapse, and second primary malignancies (SPMs) remain major barriers to both early and long-term outcomes in acute promyelocytic leukemia (APL). The prospective identification of key risk factors is essential to mitigate these adverse outcomes.

**Methods:**

We retrospectively analyzed 174 consecutive patients with newly diagnosed APL treated at a single center, with a median follow-up of 59 months. We evaluated clinical, laboratory, and treatment-related factors associated with these outcomes, including comparisons across treatment eras and regimen categories, and we summarized institutional practice regarding central nervous system (CNS) prophylaxis and management.

**Results:**

Within the cohort, ED occurred in 9 patients, relapse in 7 patients, and SPMs in 7 patients. ED clustered with severe hemorrhagic and thrombotic complications during induction. Relapse was associated with subsequent adverse outcomes, including SPMs and death. Given the low number of events, multivariable modeling was restricted and interpreted cautiously with penalized approaches used as sensitivity analyses where applicable.

**Discussion:**

Our findings support the risk-adapted approach emphasizing prompt initiation of ATRA and intensified supportive care in patients with high-risk baseline features, alongside vigilant molecular and long-term surveillance to facilitate early detection and timely management of relapse and SPMs.

## 1 Introduction

Acute promyelocytic leukemia (APL) is a rare and aggressive subtype of acute myeloid leukemia (AML). The introduction of arsenic trioxide (ATO) in the 1990s marked a significant milestone, achieving complete remission (CR) rates of 50–70% in relapsed/refractory patients and impressive overall survival (OS) in frontline therapy. Since the discovery of the PML:RARα fusion transcript and the implementation of the groundbreaking combination of all-trans retinoic acid (ATRA), ATO and chemotherapy as standard first-line treatment, survival outcomes have improved dramatically. As a result, APL has emerged as one of the most curable subtypes of AML (1), particularly among low or intermediate-risk patients. The CR rates and durable remissions was up to 95%, along with 2-year relapse rates below 10% (2).

However, despite significant advancements enabling most patients to benefit from new drugs and novel treatment combinations, challenges persist in therapeutic management and risk stratification, particularly in high-risk cases. Throughout the entire process of diagnosis, treatment and follow-up, APL faces several clinical challenges. A small but notable number of patients still die from adverse events, including early death (ED) caused by life-threatening early events during induction therapy. Furthermore, disease relapse and second primary malignancies (SPMs) threaten long-term survival, collectively posing substantial challenges to achieving a definitive cure (3–5). Currently, there is an growing recognition of the importance of adverse events, particularly the risk factors related to SPMs. Consequently, continuous analyses of various pretreatment indices and clinical manifestations that predict adverse events have become a focal point in the long-term APL management.

Despite major advances in ATRA- and ATO-based therapy, real-world evidence comparing treatment outcomes across different eras remained limited. Moreover, central nervous system (CNS) involvement in APL was poorly characterized because of its rarity and the absence of standardized prophylactic strategies. Therefore, this study evaluated the clinical and laboratory predictors of ED and SPMs, compared survival and relapse outcomes between treatment eras (2010–2017 vs. 2018–2022), and summarized institutional experience regarding the central nervous system leukemia (CNSL) prophylaxis and management. The objective was to identify significant adverse prognostic factors, establish risk-adapted therapeutic approaches, and ultimately improve both the cure rate and long-term quality of life in APL patients.

## 2 Patients and methods

### 2.1 Patients

Between February 2010 and July 2022, we conducted a single-center retrospective cohort study including 174 consecutive patients with newly diagnosed acute promyelocytic leukemia (APL) who had complete baseline clinical and laboratory information at initial presentation. The diagnosis of APL was established in accordance with the 2008 and 2016 revisions of the World Health Organization (WHO) classification. Demographic characteristics, baseline laboratory parameters, and treatment-related variables were extracted from the institutional electronic medical record system.

To examine potential temporal changes in clinical practice and outcomes, patients were categorized by calendar period into Era 0 (2010–2017) and Era 1 (2018–2022). Given the heterogeneity of induction regimens within Era 0, we prespecified an additional regimen-based classification to facilitate clinically interpretable comparisons, namely induction with ATRA plus chemotherapy without arsenic trioxide (non-ATO-based) vs. ATRA/ATO-based induction with or without concomitant chemotherapy (ATO-based). Time to ATRA initiation was defined as the interval between hospital admission and administration of the first dose of ATRA, and it was evaluated as a treatment-process indicator potentially relevant to early mortality. In addition, information regarding central nervous system (CNS) prophylaxis and CNS-directed management was abstracted, including the clinical indication, intrathecal regimen, and number of administrations.

The study protocol was reviewed and approved by the Ethics Committee of Nanjing Drum Tower Hospital. All procedures were conducted in accordance with the Declaration of Helsinki. Written informed consent was obtained from each participant in accordance with institutional requirements.

### 2.2 Definitions and endpoints

Early death (ED) was defined as all-cause mortality occurring within 30 days after hospital admission (6). Second primary malignancies (SPMs) were defined as metachronous malignant tumors diagnosed at least 6 months after the initial diagnosis of APL (7). Molecular complete remission (mCR) and relapse were defined in accordance with the revised response criteria proposed by the International Working Group (8). Central nervous system (CNS) involvement was diagnosed on the basis of the detection of leukemic promyelocytes in cerebrospinal fluid (CSF) by cytology and/or flow cytometry in conjunction with compatible neurological manifestations. Disseminated intravascular coagulation (DIC) scores were calculated according to the criteria of the International Society on Thrombosis and Haemostasis (ISTH) (9). Heart rate–corrected QT interval prolongation (QTc) was defined as > 450 ms in males or > 470 ms in females.

Differentiation syndrome (DS), previously termed retinoic acid syndrome, was defined as the occurrence of two or more of the following during induction therapy with ATRA and/or ATO: unexplained fever, weight gain, peripheral edema, dyspnea, hypotension, or acute renal failure (10, 11). The primary endpoints were ED and SPMs. Secondary endpoints included mCR, relapse (including CNS relapse), and treatment-related complications (DS, QTc prolongation, hemorrhagic events, and thrombotic events).

### 2.3 Methods

Baseline demographic, clinical, and laboratory variables were systematically collected for all patients. Demographic and clinical characteristics included sex, age, body mass index (BMI), and Eastern Cooperative Oncology Group (ECOG) performance status (12). Hematologic and biochemical parameters obtained at presentation comprised complete blood count, lactate dehydrogenase (LDH), albumin, triglycerides, cholesterol, activated partial thromboplastin time (APTT), prothrombin time (PT), fibrinogen, D-dimer, and ferritin. Morphologic evaluations were performed on peripheral blood (PB) and bone marrow (BM) smears to determine the percentages of blasts and promyelocytes.

Immunophenotyping was performed by flow cytometry and included CD2, CD5, CD7, CD11b, CD15, CD19, CD34, CD56, CD64, CD117, and HLA-DR; marker positivity was defined as expression in ≥ 20% of analyzed cells. A uniform testing protocol was applied throughout the study period to ensure comparability across calendar eras (13).

Cytogenetic and molecular assessments included conventional karyotyping, detection of the PML-RARA fusion transcript, and mutational profiling. Karyotypes were categorized as: (i) good-risk, sole *t*(15;17); (ii) normal; (iii) abnormal, *t*(15;17) with one or two additional abnormalities or other clonal abnormalities; and (iv) highly complex, *t*(15;17) with ≥ 3 additional abnormalities. Molecular profiling comprised FLT3 internal tandem duplication (FLT3-ITD) and tyrosine kinase domain (FLT3-TKD) mutations, as well as PML/RARα isoforms (bcr1, bcr2, bcr3) determined by real-time quantitative polymerase chain reaction (RT-qPCR). When available, the PML/RARα fusion transcript burden was quantified by fluorescence *in situ* hybridization (FISH).

Risk stratification was performed according to the Sanz criteria based on presenting white blood cell and platelet counts. Treatment-related complications and supportive-care–relevant variables were recorded, including disseminated intravascular coagulation (DIC) score, differentiation syndrome (DS), QTc prolongation, internal hemorrhagic events, and thrombotic events. Cerebrospinal fluid (CSF) evaluation by cytology and/or flow cytometry was performed in patients with neurological manifestations or clinical suspicion of central nervous system (CNS) involvement, and CNS-directed prophylaxis and/or therapy was documented, including intrathecal regimen and number of administrations.

### 2.4 Treatment regimens

All patients were treated in accordance with the recommendations of the Hematology Branch of the Chinese Medical Association. Induction therapy was recorded as regimen-based categories to enable clinically interpretable comparisons, including ATRA plus chemotherapy without arsenic trioxide (non-ATO-based) and ATRA/ATO-based induction with or without concomitant chemotherapy (ATO-based). In addition, patients were descriptively grouped by calendar era as Era 0 (2010–2017) and Era 1 (2018–2022) to reflect temporal changes in institutional practice.

During Era 0 (2010–2017), patients received one of the following induction regimens: (i) ATRA 20 mg/m^2^/day until complete remission (CR) in combination with anthracycline-based chemotherapy; (ii) ATRA 20 mg/m^2^/day plus arsenic trioxide (ATO) 0.16 mg/kg/day until CR; or (iii) ATRA plus ATO with the addition of anthracycline-based chemotherapy at the treating physician’s discretion. During Era 1 (2018–2022), following protocol standardization at our institution, induction predominantly consisted of ATRA 25 mg/m^2^/day plus ATO 0.16 mg/kg/day until CR. Cytoreduction with hydroxycarbamide was administered when the white blood cell (WBC) count exceeded 4 × 10^9^/L; if the WBC count increased to > 10 × 10^9^/L, idarubicin (8 mg/m^2^/day for 3 days) or daunorubicin (45 mg/m^2^/day for 3 days) was added.

Dexamethasone (10 mg intravenously twice daily) was initiated at the earliest clinical suspicion of differentiation syndrome and tapered after clinical resolution (14). Supportive care during induction was routinely provided, including transfusion support to maintain platelet counts > 40 × 10^9^/L and fibrinogen concentrations > 1.5 g/L, as well as infection prophylaxis/management, cardiac monitoring, and correction of electrolyte abnormalities.

For low- to intermediate-risk patients (WBC < 10 × 10^9^/L), consolidation consisted of seven cycles of ATRA (25 mg/m^2^/day for 2 weeks followed by a 2-week interval) and four cycles of ATO (0.16 mg/kg/day for 4 weeks followed by a 4-week interval). Maintenance therapy comprised alternating ATRA and ATO over 9 months (three 3-month cycles): ATRA was administered for 14 days during the 1st month of each cycle, followed by ATO for 14 days in the 2nd and 3rd months, each separated by 14-day intervals.

For high-risk patients (WBC ≥ 10 × 10^9^/L), consolidation included three cycles of anthracycline (idarubicin 8 mg/m^2^/day for 3 days or daunorubicin 45 mg/m^2^/day for 3 days) combined with cytarabine (Ara-C, 100 mg/m^2^/day for 5 days), followed by maintenance with alternating ATRA and ATO for approximately 2 years (eight 3-month cycles) using the same dosing schedule as for low- to intermediate-risk patients. Treatment monitoring and dose adjustments were performed according to hematologic recovery, cardiac status, and adverse event profiles.

Institutional practice regarding CNS prophylaxis was documented after remission induction. Prophylactic intrathecal therapy, when administered, typically consisted of cytarabine 50 mg, methotrexate 10 mg, and dexamethasone 5 mg. Candidates for CNS prophylaxis and the number of intrathecal administrations were determined by the treating physician on the basis of clinical risk factors (e.g., high presenting WBC count or relapse risk). For patients diagnosed with CNS involvement or relapse, CNS-directed management followed standard intrathecal treatment approaches in routine clinical practice.

### 2.5 Statistical methods

Statistical analyses were performed using SPSS version 23.0 (IBM Corp., Armonk, NY, United States) and R version 4.3.0 (packages survival, cmprsk, and survey). Continuous variables were summarized as mean ± standard deviation (SD) or median (interquartile range, IQR), as appropriate, and categorical variables were presented as counts and percentages. Between-group comparisons were conducted using the independent-samples *t*-test or the Mann-Whitney U test for continuous variables and the chi-square test or Fisher’s exact test for categorical variables.

Given the limited number of events for each endpoint, regression modeling was prespecified to be parsimonious and interpreted cautiously. Univariable logistic regression was used to examine associations with early death (ED). Variables entered into multivariable models were selected based on (i) clinical relevance supported by prior literature (e.g., WBC count/hyperleukocytosis), and/or (ii) statistical signal in univariable models (*P* < 0.10), with careful consideration of events-per-variable to avoid overfitting. rather than automated screening; penalized regression (Firth correction) was additionally applied as a sensitivity analysis to mitigate small-sample bias. Effect estimates were reported as odds ratios (ORs) with 95% confidence intervals (CIs).

To evaluate temporal differences in outcomes, patients were descriptively grouped by calendar era (Era 0: 2010–2017 vs. Era 1: 2018–2022) and, for clinically interpretable comparisons, by induction regimen category (ATO-based vs. non-ATO-based). Overall survival was estimated using the Kaplan-Meier method and compared using the log-rank test; 30-day overall survival was reported as a descriptive early outcome. For relapse and SPMs, cumulative incidence functions (CIFs) were estimated using the Aalen-Johansen estimator, with death treated as a competing event. Where comparisons between regimen/era groups were performed, inverse probability of treatment weighting (IPTW) based on propensity scores was used as a sensitivity approach to address measured baseline imbalances; covariate balance was evaluated using standardized mean differences (SMDs), with values < 0.10 indicating acceptable balance.

When applicable, Fine-Gray subdistribution hazard models were performed as sensitivity analyses to estimate subdistribution hazard ratios (sHRs) for relapse and SPMs while accounting for competing risks. Two-sided *P*-values < 0.05 were considered statistically significant.

## 3 Results

### 3.1 Baseline clinical characteristics

A total of 174 patients with APL were included in this study with the median age of 41.5 years (range, 16–75), comprising 76 males (43.7%) and 98 females (56.3%). The median follow-up time was 59.0 months. ED occurred in 9 patients (5.2%), among whom 6 (3.4%) died from DIC, 2 (1.1%) from thrombotic events (hepatic vein embolism and brainstem infarction), and 1 (0.6%) from intracranial hemorrhage.

Moreover, disease relapse occurred in 7 patients (4.0%) after the median interval of 35 months, predominantly involving the BM (*n* = 6, 3.4%), and one case of extramedullary relapse in the CNS. SPMs were identified in 7 patients (4.0%), with a median time of 51 months after APL diagnosis, including cervical cancer (*n* = 2), lung cancer (*n* = 2), papillary thyroid carcinoma (*n* = 1), gastric cancer (*n* = 1), and nasopharyngeal carcinoma (*n* = 1). Excluding the nine patients with ED, 165 patients (94.8%) achieved mCR after induction therapy.

To support temporal and regimen-based comparisons, patients were additionally summarized by calendar era (Era 0: 2010–2017, *n* = 69; Era 1: 2018–2022, *n* = 105) and by induction regimen category. Overall, ATO-based induction was administered in 131 patients (75.3%), including all 105 patients in Era 1 and 26 patients in Era 0, while non-ATO-based induction was used in 43 patients (24.7%). The median time from admission to first ATRA administration was 2.5 h (IQR, 1.8–4.2), and delayed initiation (> 6 h) was observed in 29 patients (16.7%). CNS prophylaxis with intrathecal therapy was administered in 155 patients (93.9%), with a median of 4 administrations (IQR, 3–6), typically using intrathecal cytarabine, methotrexate, and dexamethasone. Baseline clinical and laboratory features were summarized in Table 1. The baseline comparison and regression analysis of Relapse and SPMs were limited to patients who survived within 30 days and achieved mCR (*N*-16S), in order to avoid including ED cases without-long-term outcome risk in the risk set.

**TABLE 1:** Baseline clinical characteristics of the overall cohort (*N* = 174).

Variable	Total (*n* = 174)
Age (years)	41.5 (16–75)
Sex (male/female)	76/98
ECOG ≥ 2	18 (10.3%)
WBC (× 10^9^/L)	5.2 (0.7–78.4)
PLT (× 10^9^/L)	32 (8–140)
LDH (U/L)	546 (212–1810)
PT (s)	14.9 (11.0–28.6)
APTT (s)	37.5 (26.1–76.3)
Fibrinogen (g/L)	1.43 (0.38–3.67)
D-dimer (mg/L)	4.52 (0.23–35.1)
FLT3 mutation	29 (16.7%)
CD34 positivity	23 (13.2%)
CD56 positivity	18 (10.3%)
CD117 positivity	117 (67.2%)
PML-RARα isoform (bcr1/bcr3)	124/50
DIC at presentation	46 (26.4%)
High-risk group (WBC ≥ 10 × 10^9^/L)	46 (26.4%)

### 3.2 Early death

Among the 174 enrolled patients, 9 (5.2%) experienced early death (ED) within 30 days of hospital admission. The median interval from admission to death was 9 days (range, 1–26). The immediate causes of ED were predominantly coagulopathy-related complications, including disseminated intravascular coagulation (DIC) in 6 patients (66.7%), thrombotic events in 2 patients (22.2%; hepatic vein embolism and brainstem infarction), and intracranial hemorrhage in 1 patient (11.1%). ED occurred more frequently in high-risk patients (WBC ≥ 10 × 10^9^/L) than in those with low or intermediate risk (8.7% vs. 3.5%). Detailed risk-category distributions are provided in Table 2.

**TABLE 2:** Baseline clinical and laboratory characteristics: early death (ED) vs. non-ED (*N* = 174).

Unnamed: 0	ED group, *N* = 9	Non-ED group, *N* = 165	*P*-value
Sex, male [*n* (%)]	7 (77.8)	69 (41.8)	0.076^a^
Age (years), *M* (range)	24.0 (18.0, 68.0)	42.0 (16.0, 75.0)	0.110
ECOG PS ≥ 2, [*n* (%)]	8 (88.9)	72 (43.6)	0.021^a^
White blood cell count (109/L), *M* (range)	14.90 (4.30, 49.00)	2.20 (0.20, 140.20)	0.008
Hemoglobin (g/L), *M* (range)	110.0 (49.0, 129.0)	94.0 (38.0, 157.0)	0.354
Platelets (109/L), *M* (range)	17.0 (5.0, 95.0)	28.0 (4.0, 157.0)	0.302
LDH (U/L), *M* (range)	560.0 (264.0, 4679.0)	282.0 (115.0, 2823.0)	0.002
Albumin (g/L), *M* (range)	41.2 (36.7, 50.2)	40.5 (27.7, 49.2)	0.499
Triglyceride (mmol/L), *M* (range)	2.04 (1.28, 6.35)	1.67 (0.53, 7.13)	0.244
Cholesterol (mmol/L), *M* (range)	4.01 (2.88, 5.90)	4.23 (1.18, 8.87)	0.995
APTT (s), *M* (range)	27.6 (23.1, 40.8)	26.8 (17.0, 59.2)	0.219
PT (s), *M* (range)	15.6 (14.1, 23.0)	13.4 (9.8, 23.1)	0.001
Fibrinogen (g/L), *M* (range)	0.70 (0.40, 1.20)	1.30 (0.20, 5.40)	0.001
D-dimer (mg/L), *M* (range)	30.17 (0.75, 77.23)	6.60 (0.10, 111.00)	0.064
Promyelocytic ratio (bone marrow), *M* (range)	82.0 (56.0, 92.5)	85.0 (20.5, 99.0)	0.757
Promyelocytic ratio (peripheral blood), *M* (range)	91.0 (52.0, 96.0)	38.0 (0.0, 98.0)	0.001
CD2 positive, [*n* (%)]	2 (22.2)	13 (7.9)	0.377^a^
CD7 positive, [*n* (%)]	2 (22.2)	8 (4.8)	0.148^a^
CD11b positive, [*n* (%)]	2 (22.2)	17 (10.3)	0.570^a^
CD15 positive, [*n* (%)]	0 (0.0)	22 (13.3)	0.605^b^
CD19 positive, [*n* (%)]	0 (0.0)	5 (3.0)	1.000^b^
CD34 positive, [*n* (%)]	1 (11.1)	25 (15.2)	1.000^a^
CD56 positive, [*n* (%)]	2 (22.2)	15 (9.1)	0.474^a^
CD64 positive, [*n* (%)]	4 (44.4)	78 (47.3)	1.000^a^
CD117 positive, [*n* (%)]	6 (66.7)	153 (92.7)	0.035^a^
HLA-DR positive, [*n* (%)]	2 (22.2)	14 (8.5)	0.426^a^
Chromosomal karyotype, [*n* (%)]			0.214
Good	5 (55.6)	128 (77.6)	
Normal	1 (11.1)	7 (4.2)	
Abnormal	1 (11.1)	20 (12.1)	
Highly complex	2 (22.2)	10 (6.1)	
Risk group, [*n* (%)]			0.243
Low-risk	1 (11.1)	45 (27.3)	
Intermediate-risk	3 (33.3)	74 (44.8)	
High-risk	5 (55.6)	46 (27.9)	
FLT3 mutation, [*n* (%)]			< 0.001^a^
Negative	2 (22.2)	131 (79.4)	
Positive	7 (77.8)	34 (20.6)	
PML/RARα heteromer type, [*n* (%)]			0.106
1	3 (33.3)	88 (53.3)	
2	0 (0.0)	21 (12.7)	
3	6 (66.7)	56 (33.9)	
Fusion rate, *M* (range)	89.0 (28.0, 98.0)	80.0 (1.0, 98.2)	0.467
FAB type, [*n* (%)]			0.330 0.131
3a	7 (77.8)	124 (75.2)	
3b	1 (11.1)	37 (22.4)	
3v	1 (11.1)	4 (2.4)	
BMI (kg/m^2^), *M* (range)	25.01 (21.37, 30.27)	23.12 (16.60, 34.37)	
DIC score, [*n* (%)]			1.000^b^
< 5	0 (0.0)	15 (9.1)	
≥ 5	9 (100.0)	150 (90.0)	

*^a^*Continuously corrected Chi-square test;

*^b^*Fisher’s exact test.

Baseline clinical and laboratory characteristics differed between patients with ED and those who survived beyond 30 days. In univariate comparisons, ED was associated with poorer performance status, higher leukocyte burden, and more pronounced coagulation abnormalities. Specifically, patients with ED had higher ECOG scores, higher WBC counts, and higher LDH levels, together with prolonged prothrombin time and lower fibrinogen concentrations. In addition, ED cases more frequently exhibited FLT3 mutations and lower CD117 positivity. Complete univariate comparisons are summarized in Table 2.

To characterize the clinical context in which ED occurred, treatment-related adverse events were grouped by timing as early events (differentiation syndrome, QTc prolongation, internal hemorrhage, and thrombosis) and late events (SPMs, relapse, and death). Internal hemorrhage and thrombosis occurred more frequently among patients with ED, whereas differentiation syndrome and QTc prolongation were not materially different between ED and non-ED groups. These comparisons are presented in Supplementary Table 1. Consistent with the clinical course of induction, most ED events accumulated during the first 2 weeks after admission.

Given the limited number of ED events, multivariable analyses were restricted and interpreted cautiously. In a parsimonious model incorporating clinically plausible covariates, low fibrinogen concentration and FLT3 mutation remained associated with ED. These associations should be regarded as hypothesis-generating and warrant validation in larger cohorts.

### 3.3 Disease relapse

During follow-up, 7 patients (4.0%) experienced disease relapse after achieving molecular complete remission (mCR). The median time to relapse was 35 months (range, 12–68). Relapse most commonly involved the bone marrow (*n* = 6, 85.7%), whereas 1 patient (14.3%) presented with isolated extramedullary relapse involving the central nervous system (CNS).

Baseline comparisons between the relapse and non-relapse groups indicated differences in coagulation and immunophenotypic profiles. In univariate analyses, relapse was associated with prolonged activated partial thromboplastin time (APTT) and higher proportions of CD34-positive and CD56-positive leukemic cells. Other baseline characteristics, including age, sex, ECOG performance status, and fibrinogen concentration, were not materially different between groups. Detailed comparisons are provided in Table 3.

**TABLE 3:** Baseline clinical and laboratory characteristics: relapse vs. non-relapse (*N* = 165; excluding ED).

Unnamed: 0	Disease relapse group, *N* = 7	Non-disease relapse, *N* = 158	*P*-value
Sex, male [*n* (%)]	5 (71.4)	64 (40.5)	0.218^a^
Age (years), *M* (range)	53.0 (18.0, 73.0)	41.5 (16.0, 75.0)	0.174
ECOG PS ≥ 2, [*n* (%)]	4 (57.1)	68 (43.0)	0.729^a^
White blood cell count (109/L), *M* (range)	8.60 (0.60, 41.90)	2.15 (0.20, 140.20)	0.201
Hemoglobin (g/L), *M* (range)	77.0 (50.0, 129.0)	94.0 (38.0, 157.0)	0.414
Platelets (109/L), *M* (range)	18.0 (9.0, 63.0)	29.5 (4.0, 157.0)	0.433
LDH (U/L), *M* (range)	406.0 (206.0, 1258.0)	278.5 (115.0, 2823.0)	0.148
Albumin (g/L), *M* (range)	41.8 (36.8, 48.3)	40.5 (27.7, 49.2)	0.398
Triglyceride (mmol/L), *M* (range)	2.00 (0.74, 2.98)	1.67 (0.53, 7.13)	0.872
Cholesterol (mmol/L), *M* (range)	4.56 (3.43, 4.99)	4.23 (1.18, 8.87)	0.484
APTT (s), *M* (range)	29.4 (24.9, 38.2)	26.7 (17.0, 59.2)	0.037
PT (s), *M* (range)	14.2 (12.6, 18.4)	13.4 (9.8, 23.1)	0.157
Fibrinogen (g/L), *M* (range)	1.60 (0.60, 3.20)	1.30 (0.20, 5.40)	0.796
D-dimer (mg/L), *M* (range)	6.50 (3.00, 37.48)	6.61 (0.10, 111.00)	0.704
Promyelocytic ratio (bone marrow), *M* (range)	87.5 (65.0, 96.0)	85.0 (20.5, 99.0)	0.651
Promyelocytic ratio (peripheral blood), *M* (range)	62.0 (8.0, 93.0)	38.0 (0.0, 98.0)	0.327
CD2 positive, [*n* (%)]	1 (14.3)	12 (7.6)	1.000^a^
CD7 positive, [*n* (%)]	0 (0.0)	8 (5.1)	1.000^b^
CD11b positive, [*n* (%)]	0 (0.0)	17 (10.8)	1.000^b^
CD15 positive, [*n* (%)]	1 (14.3)	21 (13.3)	1.000^a^
CD19 positive, [*n* (%)]	0 (0.0)	5 (3.2)	1.000^b^
CD34 positive, [*n* (%)]	6 (85.7)	19 (12.0)	< 0.001^a^
CD56 positive, [*n* (%)]	3 (42.9)	12 (7.6)	0.012^a^
CD64 positive, [*n* (%)]	2 (28.6)	76 (48.1)	0.531^a^
CD117 positive, [*n* (%)]	6 (85.7)	147 (93.0)	1.000^a^
HLA-DR positive, [*n* (%)]	0 (0.0)	14 (8.9)	1.000^b^
Chromosomal karyotype, [*n* (%)]			0.224
Good	4 (57.1)	124 (78.5)	
Normal	0 (0.0)	7 (4.4)	
Abnormal	2 (28.6)	18 (11.4)	
Highly complex	1 (14.3)	9 (5.7)	
Risk group, [*n* (%)]			0.331
Low-risk	0 (0.0)	45 (28.5)	
Intermediate-risk	4 (57.1)	70 (44.3)	
High-risk	3 (42.9)	43 (27.2)	
FLT3 mutation, [*n* (%)]			0.347^b^
Negative	7 (100.0)	124 (78.5)	
Positive	0 (0.0)	34 (21.5)	
PML/RARα heteromer type, [*n* (%)]			0.076
1	1 (14.3)	87 (55.1)	
2	1 (14.3)	20 (12.7)	
3	5 (71.4)	51 (32.3)	
Fusion rate, *M* (range)	87.0 (79.0, 95.0)	78.5 (1.0, 98.2)	0.114
FAB type, [*n* (%)]			0.742
3a	6 (85.7)	118 (74.7)	
3b	1 (14.3)	36 (22.8)	
3v	0 (0.0)	4 (2.5)	
BMI (kg/m^2^), *M* (range)	24.14 (21.51, 28.03)	23.03 (16.60, 34.37)	0.213
DIC score, [*n* (%)]			1.000^b^
<5	0 (0.0)	15 (9.5)	
≥ 5	7 (100.0)	143 (90.5)	

*^a^*Continuously corrected Chi-square test;

*^b^*Fisher’s exact test.

When treatment-related adverse events were examined by timing, the incidence of early events—differentiation syndrome, QTc prolongation, internal hemorrhage, and thrombosis—did not differ significantly between relapse and non-relapse subgroups. By contrast, patients who relapsed showed higher frequencies of late adverse outcomes, including SPMs and death, during subsequent follow-up. These event comparisons are summarized in Supplementary Table 2. In addition, the time to achieve mCR was longer in patients who later relapsed, suggesting that delayed molecular response co-occurred with relapse risk in this cohort.

To incorporate the treatment-era comparison, relapse outcomes were further summarized by calendar era and regimen category. The cumulative incidence of relapse was lower in Era 1 than in Era 0, as illustrated by the competing-risk cumulative incidence curves. Era- and regimen-stratified relapse estimates were summarized. Given the small number of relapse events, multivariable modeling was restricted and interpreted conservatively. In parsimonious models, CD34 and CD56 positivity remained associated with relapse.

### 3.4 Second primary malignancies

During follow-up, 7 patients (4.0%) developed second primary malignancies (SPMs) after achieving sustained molecular remission. The median latency from APL diagnosis to SPM occurrence was 51 months (range, 27–93). The SPM spectrum comprised cervical cancer (*n* = 2), lung cancer (*n* = 2), papillary thyroid carcinoma (*n* = 1), gastric cancer (*n* = 1), and nasopharyngeal carcinoma (*n* = 1). All SPMs were histopathologically confirmed, and no hematologic secondary malignancies were observed.

**FIGURE 1:**
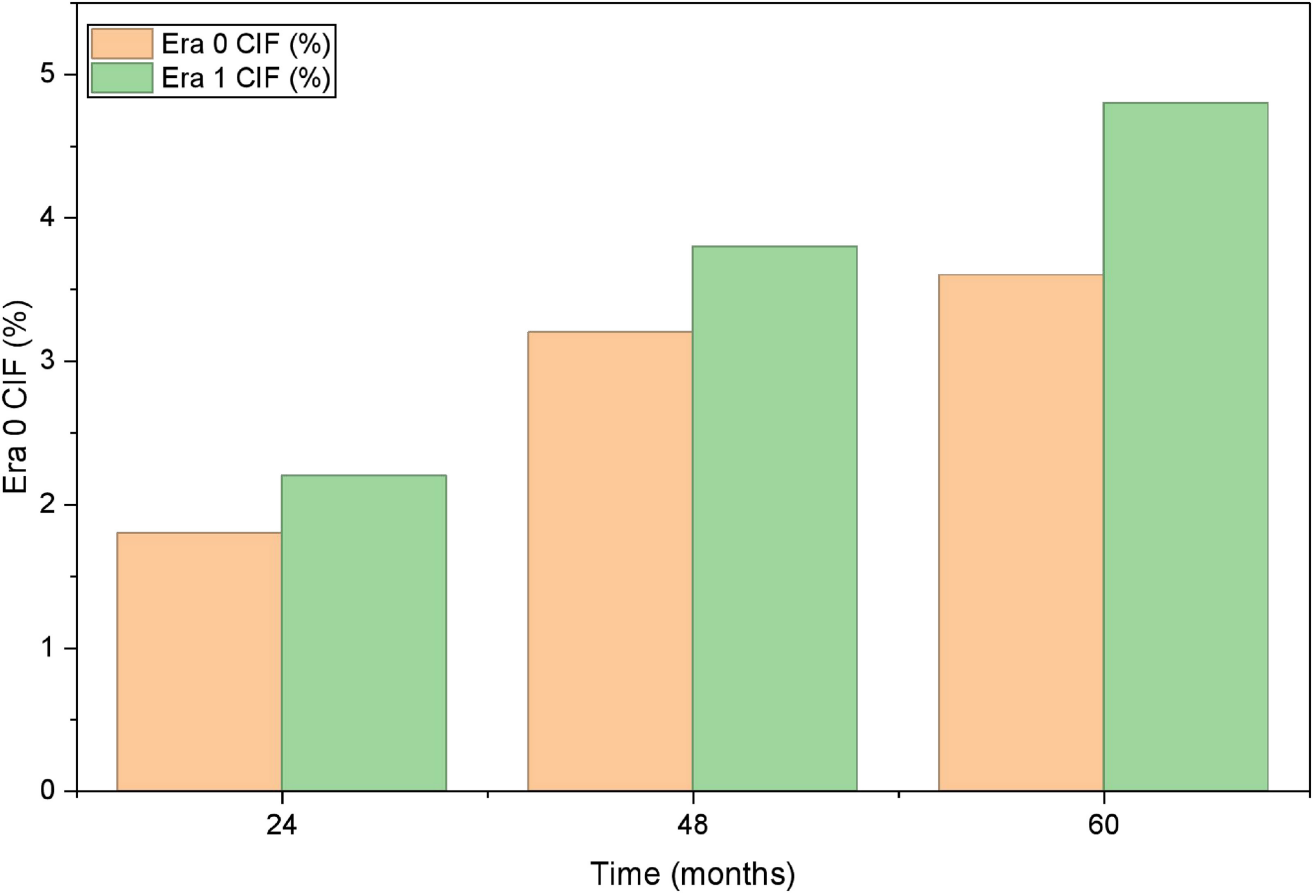
Cumulative incidence function for SPMs.

Baseline clinical and laboratory characteristics were comparable between patients who developed SPMs and those who did not, with no statistically significant differences detected in the measured variables (Table 4). Similarly, the incidence of early adverse events—including differentiation syndrome, QTc prolongation, internal hemorrhage, and thrombosis—did not differ materially between groups. By contrast, patients who developed SPMs more frequently experienced subsequent relapse and all-cause mortality during follow-up than those without SPMs (Supplementary Table 3). The time to achieve mCR was not significantly different between the two groups. Given the low number of SPM events, regression-based competing-risk analyses were considered exploratory; therefore, SPM-related associations with other late outcomes are presented primarily as descriptive comparisons, with additional exploratory models provided in Supplementary material. When stratified by calendar era, the 60-month cumulative incidence of SPMs was similar between Era 0 (2010–2017) and Era 1 (2018–2022), as shown by the cumulative incidence curves (Figure 1) and summarized.

**TABLE 4:** Baseline clinical and laboratory characteristics: second primary malignancies (SPMs) vs. non-SPMs (*N* = 165; excluding ED).

Unnamed: 0	SPMs group, *N* = 7	Non-SPMs group, *N* = 158	*P*-value
Sex, male [*n* (%)]	2 (28.6)	67 (42.4)	0.738^a^
Age (years), *M* (range)	51.0 (22.0, 73.0)	41.5 (16.0, 75.0)	0.627
ECOG PS ≥ 2, [*n* (%)]	3 (42.9)	69 (43.7)	1.000^a^
White blood cell count (109/L), *M* (range)	1.50 (0.60, 42.0)	2.25 (0.20, 140.20)	0.599
Hemoglobin (g/L), *M* (range)	91.0 (77.0, 129.0)	94.0 (38.0, 157.0)	0.740
Platelets (109/L), *M* (range)	37.0 (15.0, 41.0)	28.0 (4.0, 157.0)	0.683
LDH (U/L), *M* (range)	252.0 (206.0, 406.0)	284.0 (115.0, 2823.0)	0.731
Albumin (g/L), *M* (range)	43.0 (37.2, 48.3)	40.4 (27.7, 49.2)	0.185
Triglyceride (mmol/L), *M* (range)	1.47 (0.80, 2.10)	1.67 (0.53, 7.13)	0.400
Cholesterol (mmol/L), *M* (range)	4.72 (4.10, 5.10)	4.22 (1.18, 8.87)	0.194
APTT (s), *M* (range)	28.8 (22.4, 39.9)	26.8 (17.0, 59.2)	0.270
PT (s), *M* (range)	13.4 (12.6, 14.6)	13.5 (9.8, 23.1)	0.746
Fibrinogen (g/L), *M* (range)	1.60 (0.90, 3.40)	1.30 (0.20, 5.40)	0.118
D-dimer (mg/L), *M* (range)	6.60 (3.00, 37.52)	6.59 (0.10, 111.00)	0.383
Promyelocytic ratio (bone marrow), *M* (range)	80.5 (65.0, 87.5)	85.3 (20.5, 99.0)	0.111
Promyelocytic ratio (peripheral blood), *M* (range)	24.0 (0.0, 93.0)	40.0 (0.0, 98.0)	0.739
CD2 positive, [*n* (%)]	1 (14.3)	12 (7.6)	1.000^a^
CD7 positive, [*n* (%)]	0 (0.0)	8 (5.1)	1.000^b^
CD11b positive, [*n* (%)]	1 (14.3)	16 (10.1)	1.000^a^
CD15 positive, [*n* (%)]	1 (14.3)	21 (13.3)	1.000^a^
CD19 positive, [*n* (%)]	0 (0.0)	5 (3.2)	1.000^b^
CD34 positive, [*n* (%)]	2 (28.6)	23 (14.6)	0.636^a^
CD56 positive, [*n* (%)]	2 (28.6)	13 (8.2)	0.246^a^
CD64 positive, [*n* (%)]	3 (42.9)	75 (47.5)	1.000^a^
CD117 positive, [*n* (%)]	7 (100.0)	146 (92.4)	1.000^b^
HLA-DR positive, [*n* (%)]	0 (0.0)	14 (8.9)	1.000^b^
Chromosomal karyotype, [*n* (%)]			0.224
Good	4 (57.1)	124 (78.5)	
Normal	0 (0.0)	7 (4.4)	
Abnormal	3 (42.9)	17 (10.8)	
Highly complex	0 (0.0)	10 (6.3)	
Risk group, [*n* (%)]			0.416
Low-risk	1 (14.3)	44 (27.8)	
Intermediate-risk	5 (71.4)	69 (43.7)	
High-risk	1 (14.3)	45 (28.5)	
FLT3 mutation, [*n* (%)]			1.000^a^
Negative	6 (85.7)	125 (79.1)	
Positive	1 (14.3)	33 (20.9)	
PML/RARα heteromer type, [*n* (%)]			0.760 0.531
1	4 (57.1)	84 (53.2)	
2	0 (0.0)	21 (13.3)	
3	3 (42.9)	53 (33.5)	
Fusion rate, *M* (range)	85.5 (70.5, 91.5)	80.0 (1.0, 98.2)	
FAB type, [*n* (%)]			0.742
3a	6 (85.7)	118 (74.7)	
3b	1 (14.3)	36 (22.8)	
3v	0 (0.0)	4 (2.5)	
BMI (kg/m^2^), *M* (range)	23.62 (17.19, 28.03)	23.12 (16.60, 34.37)	0.916
DIC score, [*n* (%)]			1.000^b^
<5	0 (0.0)	15 (9.5)	
≥ 5	7 (100.0)	143 (90.5)	

*^a^*Continuously corrected Chi-square test;

*^b^*Fisher’s exact test.

### 3.5 Prognostic analysis

Prognostic analyses were performed to examine baseline and treatment-related factors associated with ED, relapse, and SPMs. Given the low number of events for each endpoint, effect estimates were interpreted conservatively, and regression models were restricted to reduce the risk of overfitting. Detailed results are provided in Supplementary Tables 4–6.

#### 3.5.1 Univariate analyses

In univariable logistic regression, several clinical and laboratory variables were associated with ED. These included ECOG performance status ≥ 2 (OR 10.33, 95% CI 1.26.33, 95re associated with ED. These included ECOG pe-dimer, increased peripheral promyelocyte percentage, CD117 positivity, and FLT3 mutation (Supplementary Table 4). For relapse, CD34 positivity and CD56 positivity showed strong associations with relapse occurrence in univariable analyses (Supplementary Table 4). No baseline variable reached statistical significance for SPMs in univariable analyses.

#### 3.5.2 Multivariate analyses

To minimize overfitting, multivariable models were specified parsimoniously, incorporating a limited set of clinically plausible covariates rather than automated stepwise selection. In these restricted models, low fibrinogen and FLT3 mutation remained associated with ED, whereas CD34 and CD56 positivity remained associated with relapse (Supplementary Tables 5, 6). No independent association was identified for SPMs in multivariable analyses. These multivariable results should be regarded as hypothesis-generating given the limited number of events.

#### 3.5.3 Time-to-event and competing-risk analyses

**FIGURE 2:**
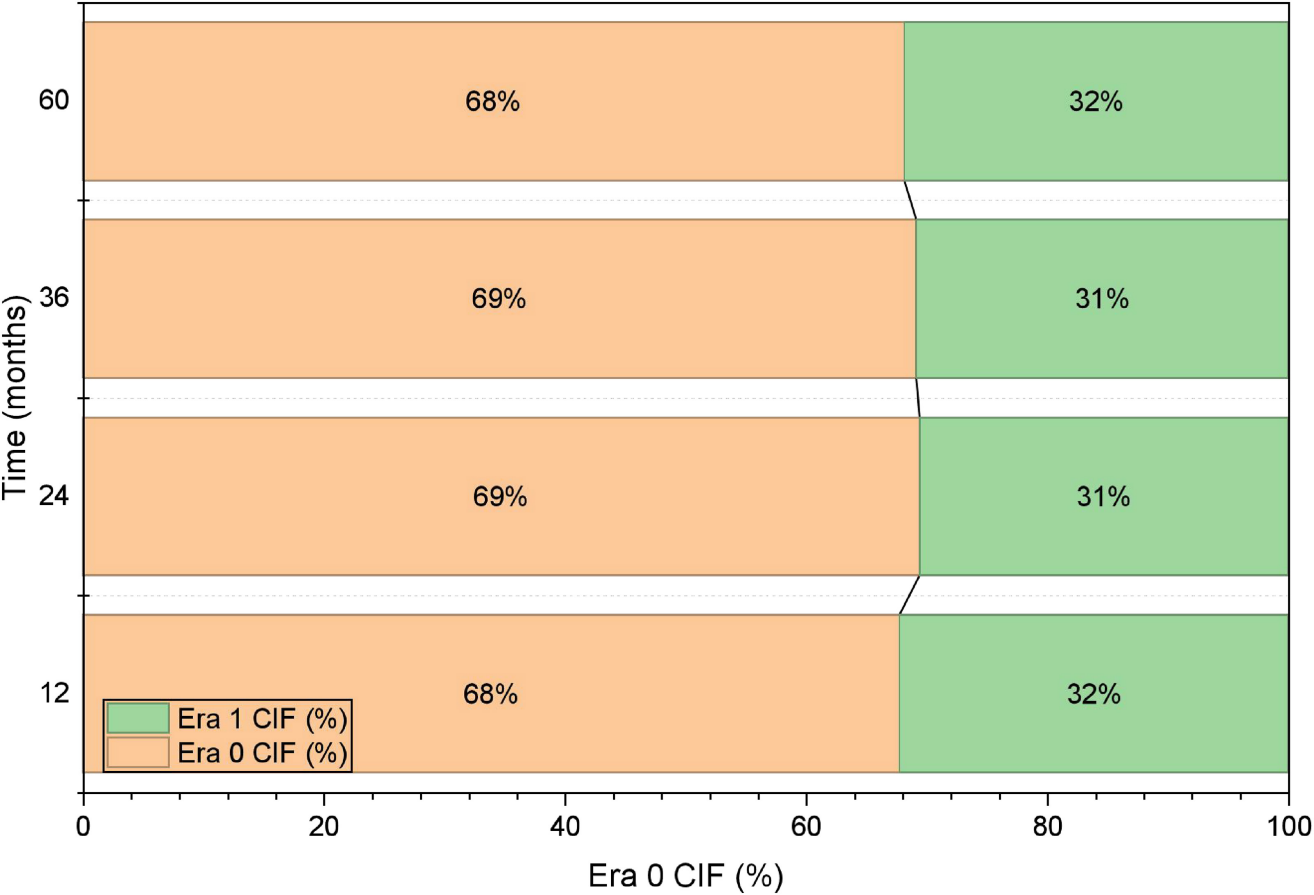
Cumulative incidence function for relapse.

**FIGURE 3:**
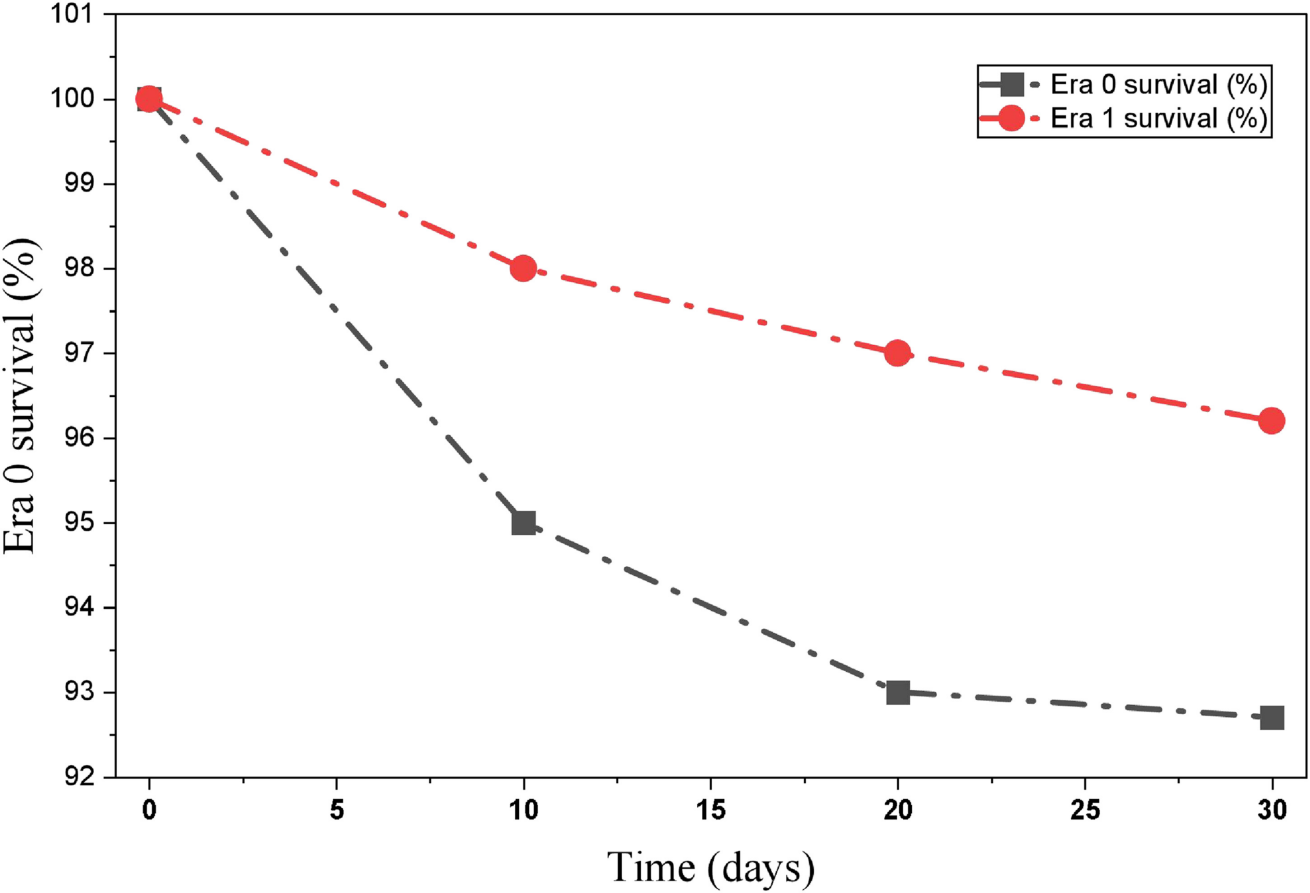
Kaplan-Meier (30-day survival).

Time-to-event analyses were presented primarily using Kaplan-Meier estimates for early survival and cumulative incidence functions (CIFs) for relapse and SPMs with death treated as a competing event (Figures 1–3). Competing-risk regression (Fine-Gray subdistribution hazard models) was performed as an exploratory sensitivity analysis to evaluate whether the direction of associations was consistent in a time-to-event framework. In these exploratory models, CD34/CD56 positivity showed an elevated subdistribution hazard for relapse, and FLT3 mutation and low fibrinogen showed elevated hazards for ED. However, the precision of these estimates was limited by the small number of events, and the corresponding effect sizes should be interpreted cautiously. No factor demonstrated a statistically robust association with SPMs in the competing-risk framework.

#### 3.5.4 Era-stratified outcomes and weighting-based sensitivity analyses

**FIGURE 4:**
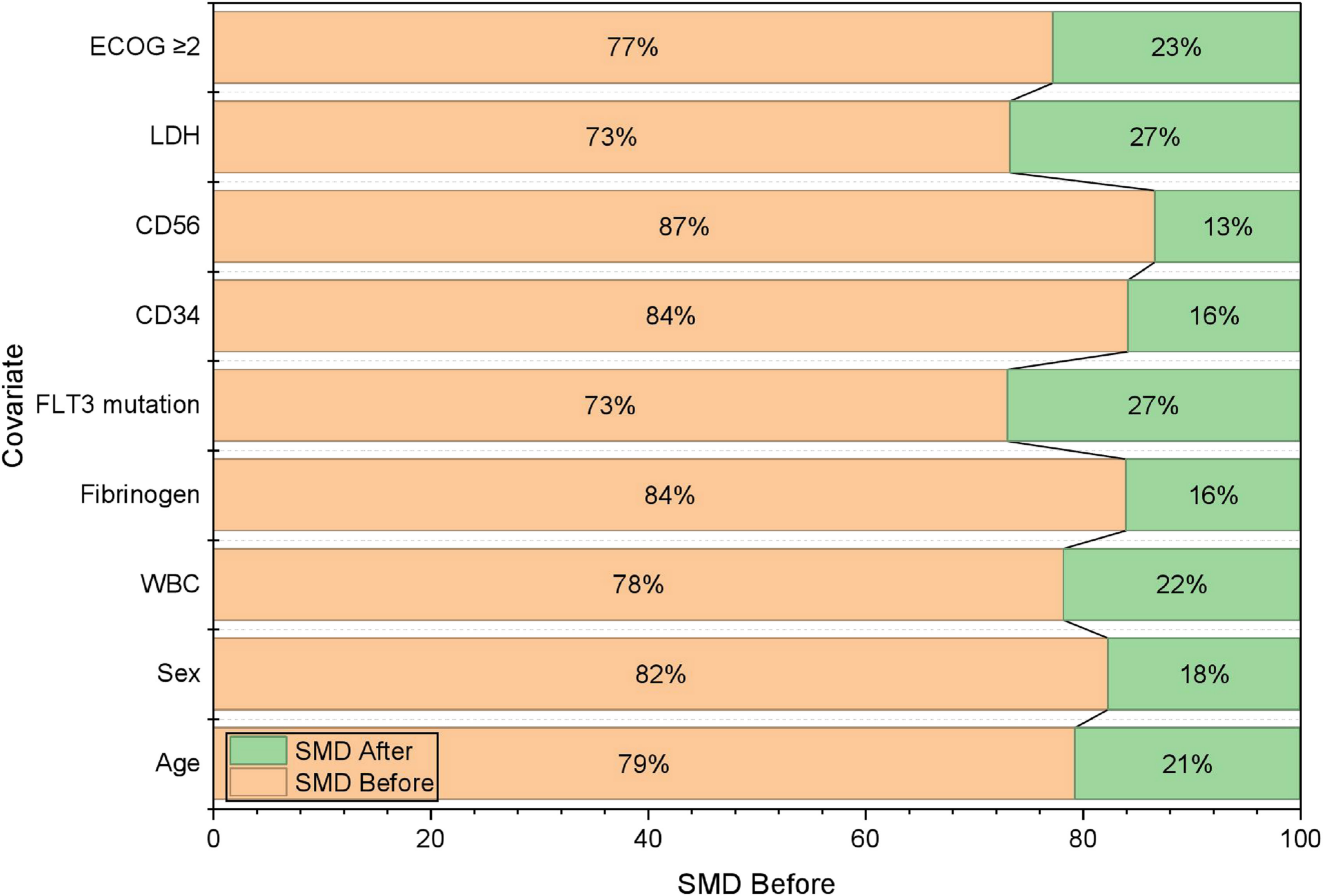
IPTW covariate balance (love plot).

Outcomes were further summarized by calendar era. The 30-day ED rate was lower in Era 1 than in Era 0, and relapse incidence was also lower in Era 1, whereas the cumulative incidence of SPMs was comparable between eras (Table 5). To address baseline imbalances between eras, IPTW was applied as a sensitivity approach, and covariate balance was assessed using standardized mean differences; post-weighting balance metrics are shown in Figure 4. These analyses were intended to provide complementary, descriptive evidence for temporal changes in outcomes under evolving practice patterns, rather than definitive causal inference.

**TABLE 5:** Treatment-era comparison of key outcomes (Era 0 vs. Era 1; *N* = 174).

Outcome	Era 0 (2010–2017, *n* = 69)	Era 1 (2018–2022, *n* = 105)	*P*-value
Early death (ED)	5 (7.3%)	4 (3.8%)	0.046
Relapse	4 (5.8%)	3 (2.9%)	0.041
SPMs	2 (3.6%)	5 (4.8%)	0.72
30-day survival	92.7%	96.2%	0.046
60-month relapse incidence	6.2%	2.9%	0.041
60-month SPM incidence	3.6%	4.8%	0.72
IPTW SMD (all covariates)	< 0.1	<0.1	–

### 3.6 Treatment-era comparison

To describe temporal changes in outcomes under evolving institutional practice, patients were grouped into two calendar eras: Era 0 (2010–2017, *n* = 69), during which induction regimens were heterogeneous, and Era 1 (2018–2022, *n* = 105), during which a standardized ATRA + ATO-based induction and consolidation protocol predominated.

#### 3.6.1 Early outcomes

Thirty-day overall survival was higher in Era 1 than in Era 0 (92.7% vs. 96.2%), as shown by Kaplan-Meier estimates (Figure 3). Consistently, the proportion of patients experiencing ED was lower in Era 1 (7.3% vs. 3.8%). Because baseline characteristics differed between eras, IPTW was applied as a sensitivity approach to improve covariate balance; post-weighting standardized mean differences were < 0.1 across measured covariates. In an exploratory adjusted analysis treating era as a covariate, Era 1 was associated with lower odds of ED; however, given the limited number of ED events, this estimate was interpreted cautiously and considered hypothesis-generating.

#### 3.6.2 Relapse and long-term outcomes

Relapse incidence differed between eras in time-to-event analyses. Using the Aalen-Johansen estimator with death treated as a competing event, the 5-year cumulative incidence of relapse was lower in Era 1 than in Era 0 (Figure 2). Exploratory competing-risk regression (Fine-Gray) yielded a directionally consistent association between Era 1 and lower relapse risk, although the precision of effect estimates was limited by the small number of relapse events. The observed time to relapse also tended to be longer in Era 1, whereas the median time to relapse in Era 1 was not reached within available follow-up.

#### 3.6.3 Second primary malignancies

The cumulative 60-month incidence of SPMs was similar between Era 0 and Era 1 (3.6% vs. 4.8%), with overlapping cumulative incidence curves. Median latency to SPM onset was comparable between eras. No clear temporal difference in the distribution of SPM types was observed in this cohort (Figure 1).

#### 3.6.4 Impact on prognostic determinants

To explore whether the strength of associations for selected prognostic variables differed by era, era-stratified analyses were performed. The associations of FLT3 mutation and low fibrinogen with ED appeared weaker in Era 1 than in Era 0; however, statistical power was limited and these comparisons were considered exploratory. In contrast, CD34/CD56-positive immunophenotypes remained associated with relapse across eras in adjusted models, although effect estimates should be interpreted cautiously given the small number of relapse events.

Overall, outcomes for early survival and relapse were more favorable in Era 1 than in Era 0 in descriptive and time-to-event analyses, whereas SPM incidence was comparable between eras. IPTW-based balance diagnostics and competing-risk analyses were used as sensitivity approaches to complement unadjusted comparisons.

## 4 Discussion

### 4.1 Early death: persistent clinical challenge despite therapeutic progress

Early death (ED) remained the predominant cause of treatment failure in acute promyelocytic leukemia (APL). In multicenter clinical trials, ED rates were commonly reported between 5 and 10% (15–18); however, real-world series often described substantially higher incidences (17–32%), and even 24–50% among high-risk patients (4, 19). This discrepancy was plausibly attributable to differences in case mix, referral patterns, baseline leukocytosis/coagulopathy severity, and the availability of timely expertise and supportive resources. In the present cohort of 174 patients, the observed ED rate was 5.2%, which was comparatively low among real-world reports and was consistent with data suggesting reduced early mortality in urban settings and in higher-volume institutions with structured care pathways (20, 21).

Despite therapeutic advances, ED still occurred predominantly during induction and was clinically driven by hemorrhagic and thrombotic complications. Hyperleukocytosis was a well-established driver of early mortality; FLT3 mutations may contributed to ED partly through leukocytosis and aggressive disease biology. In this study, FLT3 mutation and low fibrinogen concentration independently predicted ED, supporting the concept that hyperproliferative biology and profound hemostatic dysregulation jointly contributed to early mortality (22, 23). Accordingly, patients presenting with FLT3 mutation and/or hypofibrinogenemia warranted immediate escalation of supportive care, including aggressive correction of coagulopathy, meticulous transfusion support to maintain hemostatic targets, and prompt cytoreductive control of leukocytosis when indicated. In addition, early recognition and management of differentiation syndrome (DS), infection, and cardiotoxicity surveillance remained integral to reducing preventable induction-related deaths.

Importantly, implementation of a more standardized ATRA-ATO-based protocol in the later era coincided with fewer ED events, suggesting that standardized therapy coupled with optimized supportive management contributed to improved early outcomes. Nonetheless, given the small number of ED events, the precision of effect estimates remained limited and the findings should be interpreted as hypothesis-generating, requiring validation in larger multicenter cohorts.

### 4.2 Disease relapse: prognostic relevance of CD34/CD56 and implications for individualized post-remission management

Although the introduction of ATRA and ATO markedly improved survival in APL, relapse continued to occur and remained clinically relevant (24, 25). Therefore, identifying relapse-prone subgroups was essential for risk-adapted consolidation, maintenance, and follow-up strategies. Prior studies proposed multiple relapse-associated factors, including high baseline WBC count (> 10 × 10^9^/L), additional cytogenetic abnormalities, microgranular morphology, bcr3 PML-RARA isoform, and immunophenotypic immaturity characterized by CD34/CD56 expression (26, 27).

In the present cohort, CD34 and/or CD56 positivity remained independently associated with relapse risk, aligning with their association with an immature leukemic phenotype and potential persistence of minimal residual disease. These findings supported the clinical rationale that CD34/CD56-positive patients could benefit from intensified post-remission strategies, such as reinforced consolidation intensity, prolonged maintenance, and closer molecular monitoring, as suggested in prior treatment optimization experience (28). During follow-up, serial quantification of PML–RARA transcripts provided a sensitive approach to detect molecular recurrence; once relapse was confirmed, timely initiation of salvage therapy was critical to prevent overt hematologic progression and to optimize long-term outcomes.

Notably, one relapse occurred in the central nervous system (CNS), underscoring that extramedullary relapse remained clinically meaningful. Institutional experience with CNS prophylaxis/management therefore represented a pragmatic component of real-world care, particularly in patients considered at elevated risk, and warranted clear reporting in the treatment section and discussion of its indications and limitations.

### 4.3 Second primary malignancies: long-term safety and surveillance

SPMs have emerged as a clinically relevant late complication among long-term APL survivors. Most previously reported SPMs were solid tumors, followed by myelodysplastic syndromes or therapy-related hematologic malignancies (7, 29–31). Lenzi et al. (32) identified age ≥ 40 years and non-Hispanic white ethnicity as independent risk factors for SPM development, with a cumulative incidence approaching 7% at 10 years.

In this cohort, SPMs were identified in 7 patients (4.0%) after a median latency of 51 months, with all seven cases being solid tumors. No hematologic secondary neoplasms were detected, and no treatment-related risk factors were identified—likely due to the small sample size and limited follow-up. A key finding from our data is that the cumulative 60-month SPM incidence did not differ significantly between the two treatment eras (3.6% vs. 4.8%, *P* = 0.72), indicating that the widespread adoption of ATO-based therapy and reduced anthracycline exposure did not increase carcinogenic risk. Given the expanding survivor population, continued long-term monitoring using tumor markers, imaging, and endoscopic screening remains imperative to enable early diagnosis and timely intervention.

### 4.4 Adverse clinical events and coagulopathy: hemorrhage and thrombosis as dual threats

Hemorrhagic and thrombotic events represent the dual manifestations of APL coagulopathy and remain key determinants of induction mortality (33). Our findings reaffirm that in our patient cohort, internal hemorrhage continues to be the leading cause of ED, whereas thrombosis, though less frequent, significantly compromises clinical outcomes and contributes to long-term morbidity. These complications are mechanistically linked to procoagulant microparticle release, fibrinolytic activation, and endothelial injury induced by APL promyelocytes. Thus, balanced transfusion support, early anticoagulation when indicated, and prevention of DIC exacerbation are vital for reducing induction-related mortality. Benefiting from advances in ATO-based therapy, the overall survival of APL patients has been markedly prolonged. However, the persistence of coagulation abnormalities and inflammatory activation may predispose survivors to chronic vascular injury or secondary malignancy. Future studies should evaluate whether genetic susceptibility, inflammatory burden, or prior anthracycline exposure contributes to late events, particularly relapse and SPMs.

### 4.5 Clinical implications, limitations, and future perspectives

Collectively, the present study delineated a pragmatic prognostic model in APL integrating (i) hemostatic dysregulation (low fibrinogen and related coagulopathy) as a driver of early mortality, (ii) immunophenotypic immaturity (CD34/CD56 positivity) as a determinant of relapse susceptibility, and (iii) molecular features such as FLT3 mutation as markers of aggressive biology influencing early outcomes. The era-based comparison suggested that standardization toward ATRA-ATO-based therapy coincided with improved early survival and reduced relapse without an apparent increase in SPM risk within the observed timeframe.

Several limitations should be acknowledged. First, the retrospective single-center design limited generalizability and introduced unavoidable selection and information biases. Second, the number of ED, relapse, and SPM events was small, which constrained statistical power and could compromise the stability of multivariable models, increasing the risk of overfitting and imprecise effect estimates. Accordingly, the magnitude of odds ratios and hazard estimates should be interpreted cautiously. Third, although clinical practice emphasized urgent initiation of ATRA and intensive supportive care, granular and uniformly recorded time-to-treatment metrics were not available for all patients, precluding a formal quantitative assessment of diagnostic/treatment delay effects in this cohort. Future multicenter prospective studies with standardized supportive-care metrics, longitudinal molecular monitoring, CNS surveillance strategies, and competing-risk frameworks are warranted to validate and refine risk-adapted management algorithms for APL.

## Data Availability

The original contributions presented in this study are included in this article/Supplementary material, further inquiries can be directed to the corresponding authors.
