# Supplementary material for: Risk factors associated with early death, disease relapse and second primary malignancies in patients with newly diagnosed acute promyelocytic leukemia
Source: Front Med (Lausanne). 2026 Feb 19;13:1671705. doi: 10.3389/fmed.2026.1671705 (PMC12960103; doi:10.3389/fmed.2026.1671705)
Supplement: Supplementary file 1 [file Supplementary_file_1.docx]

**Supplementary Table S1. Early adverse events: ED vs. non-ED (N = 174)**

| **Unnamed: 0** | **ED group, N=9** | **non-ED group, N=165** | **P-value** |
| --- | --- | --- | --- |
| Differentiation syndrome, [n (%)] | 4 (44.4) | 39 (23.6) | 0.311a |
| QT interval prolongation, [n (%)] | 0 (0.0) | 6 (3.6) | 1.000b |
| Internal hemorrhage, [n (%)] | 7 (77.8) | 18 (10.9) | <0.001a |
| Thrombosis, [n (%)] | 2 (22.2) | 5 (3.0) | 0.047a |

^a^continuously corrected Chi-square test; ^b^Fisher's exact test.

**Supplementary Table S2. Adverse events and time to molecular complete remission (mCR): relapse vs. non-relapse (N = 165; excluding ED)**

| **Unnamed: 0** | **disease relapse group, N=7** | **non-disease relapse group, N=158** | **P-value** |
| --- | --- | --- | --- |
| Early events, [n (%)] |  |  |  |
| Differentiation syndrome | 0 (0.0) | 39 (24.7) | 0.200b |
| QT prolongation | 0 (0.0) | 6 (3.8) | 1.000b |
| Internal hemorrhage | 2 (28.6) | 16 (10.1) | 0.362a |
| Thrombosis | 0 (0.0) | 5 (3.2) | 1.000b |
| Late events, [n (%)] |  |  |  |
| QT prolongation | 0 (0.0) | 3 (1.9) | 1.000b |
| Internal hemorrhage | 0 (0.0) | 2 (1.3) | 1.000b |
| second primary malignancy | 3 (42.9) | 4 (2.5) | <0.001a |
| Death | 5 (71.4) | 2 (1.3) | <0.001a |
| Time to mCR (days), M(range) | 46.0 (33.0, 78.0) | 38.5 (28.0, 72.0) | 0.039 |

^a^continuously corrected Chi-square test; ^b^Fisher's exact test.

**Supplementary Table S3. Adverse events and time to molecular complete remission (mCR): SPMs vs. non-SPMs (N = 165; excluding ED)**

| **Unnamed: 0** | **SPMs group, N=7** | **non-SPMs group, N=158** | **P-value** |
| --- | --- | --- | --- |
| Early events, [n (%)] |  |  |  |
| Differentiation syndrome | 0 (0.0) | 39 (24.7) | 0.200b |
| QT interval prolongation | 0 (0.0) | 6 (3.8) | 1.000b |
| Internal bleeding | 0 (0.0) | 18 (11.4) | 0.608b |
| Thrombosis | 0 (0.0) | 5 (3.2) | 1.000b |
| Late events, [n (%)] |  |  |  |
| QT interval prolongation | 0 (0.0) | 3 (1.9) | 1.000b |
| Internal bleeding | 0 (0.0) | 2 (1.3) | 1.000b |
| Disease relapse | 3 (42.9) | 4 (2.5) | <0.001a |
| Death | 2 (28.6) | 5 (3.2) | 0.021a |
| Time to mCR (days), M(range) | 48.0 (30.0, 75.0) | 38.5 (28.0, 78.0) | 0.131 |

^a^continuously corrected Chi-square test; ^b^Fisher's exact test.

**Supplementary Table S4. Univariable logistic regression analyses for ED, relapse, and SPMs**

| **Unnamed: 0** | **early death events** | **early death events.1** | **disease relapse events** | **disease relapse events.1** | **second primary malignancy events** | **second primary malignancy events.1** | **second primary malignancy events.2** | **Unnamed: 8** |
| --- | --- | --- | --- | --- | --- | --- | --- | --- |
|  | P-value | HR (95%CI) | P-value | HR (95%CI) | HR (95%CI) | P-value | HR (95%CI) | HR (95%CI) |
| Sex, female | 0.053 | 0.205 (0.041-1.019) | 0.127 | 0.272 (0.051-1.447) | 0.272 (0.051-1.447) | 0.474 | 1.841 (0.347-9.777) | 1.841 (0.347-9.777) |
| Age (years) | 0.181 | 0.965 (0.917-1.017) | 0.164 | 1.040 (0.984-1.099) | 1.040 (0.984-1.099) | 0.443 | 1.021 (0.968-1.077) | 1.021 (0.968-1.077) |
| ECOG PS≥2 | 0.029 | 10.333 (1.263-84.510) | 0.467 | 1.765 (0.382-8.147) | 1.765 (0.382-8.147) | 0.966 | 0.967 (0.210-4.466) | 0.967 (0.210-4.466) |
| White blood cell count (109/L) | 0.550 | 1.007 (0.984-1.030) | 0.813 | 1.003 (0.976-1.032) | 1.003 (0.976-1.032) | 0.580 | 0.986 (0.939-1.036) | 0.986 (0.939-1.036) |
| Hemoglobin (g/L) | 0.440 | 1.010 (0.985-1.036) | 0.354 | 0.986 (0.957-1.016) | 0.986 (0.957-1.016) | 0.712 | 1.005 (0.977-1.035) | 1.005 (0.977-1.035) |
| Platelets (109/L) | 0.557 | 0.992 (0.968-1.018) | 0.351 | 0.983 (0.949-1.019) | 0.983 (0.949-1.019) | 0.626 | 0.993 (0.965-1.021) | 0.993 (0.965-1.021) |
| LDH (U/L) | 0.018 | 1.002 (1.000-1.003) | 0.347 | 1.001 (0.999-1.003) | 1.001 (0.999-1.003) | 0.331 | 0.998 (0.993-1.002) | 0.998 (0.993-1.002) |
| Albumin (g/L) | 0.322 | 1.093 (0.917-1.304) | 0.314 | 1.110 (0.906-1.361) | 1.110 (0.906-1.361) | 0.162 | 1.164 (0.941-1.440) | 1.164 (0.941-1.440) |
| Triglyceride (mmol/L) | 0.280 | 1.288 (0.813-2.041) | 0.744 | 0.884 (0.420-1.857) | 0.884 (0.420-1.857) | 0.292 | 0.572 (0.203-1.615) | 0.572 (0.203-1.615) |
| Cholesterol (mmol/L) | 0.879 | 1.050 (0.560-1.969) | 0.704 | 1.143 (0.574-2.275) | 1.143 (0.574-2.275) | 0.401 | 1.322 (0.689-2.540) | 1.322 (0.689-2.540) |
| APTT(s) | 0.119 | 1.079 (0.980-1.188) | 0.101 | 1.089 (0.983-1.206) | 1.089 (0.983-1.206) | 0.210 | 1.071 (0.962-1.193) | 1.071 (0.962-1.193) |
| PT(s) | 0.002 | 1.521 (1.174-1.972) | 0.141 | 1.262 (0.926-1.721) | 1.262 (0.926-1.721) | 0.549 | 0.877 (0.571-1.347) | 0.877 (0.571-1.347) |
| Fibrinogen (g/L) | 0.011 | 0.056 (0.006-0.513) | 0.883 | 1.062 (0.476-2.367) | 1.062 (0.476-2.367) | 0.193 | 1.553 (0.800-3.013) | 1.553 (0.800-3.013) |
| D-dimer (mg/L) | 0.029 | 1.031 (1.003-1.059) | 0.760 | 1.007 (0.964-1.051) | 1.007 (0.964-1.051) | 0.682 | 1.009 (0.968-1.051) | 1.009 (0.968-1.051) |
| Promyelocytic ratio (bone marrow) | 0.882 | 1.004 (0.956-1.054) | 0.588 | 1.018 (0.955-1.085) | 1.018 (0.955-1.085) | 0.433 | 0.983 (0.941-1.026) | 0.983 (0.941-1.026) |
| Promyelocytic ratio (peripheral blood) | 0.010 | 1.046 (1.011-1.082) | 0.404 | 1.009 (0.987-1.032) | 1.009 (0.987-1.032) | 0.624 | 0.994 (0.972-1.017) | 0.994 (0.972-1.017) |
| CD2 positive | 0.157 | 3.341 (0.629-17.753) | 0.528 | 2.028 (0.225-18.251) | 2.028 (0.225-18.251) | 0.528 | 2.208 (0.225-18.251) | 2.208 (0.225-18.251) |
| CD7 positive | 0.050 | 5.607 (0.999-31.457) | 0.999 | - | - | 0.999 | - | - |
| CD11b positive | 0.279 | 2.487 (0.478-12.948) | 0.999 | - | - | 0.725 | 1.479 (0.167-13.074) | 1.479 (0.167-13.074) |
| CD15 positive | 0.998 | - | 0.940 | 1.087 (0.125-9.487) | 1.087 (0.125-9.487) | 0.940 | 1.087 (0.125-9.487) | 1.087 (0.125-9.487) |
| CD19 positive | 0.999 | - | 0.999 | - | - | 0.999 | - | - |
| CD34 positive | 0.742 | 0.700 (0.084-5.843) | 0.001 | 43.895 (5.009-384.675) | 43.895 (5.009-384.675) | 0.325 | 2.348 (0.430-12.831) | 2.348 (0.430-12.831) |
| CD56 positive | 0.215 | 2.857 (0.544-15.007) | 0.007 | 9.125 (1.827-45.582) | 9.125 (1.827-45.582) | 0.091 | 4.462 (0.787-25.298) | 4.462 (0.787-25.298) |
| CD64 positive | 0.869 | 0.892 (0.231-3.441) | 0.324 | 0.432 (0.081-2.291) | 0.432 (0.081-2.291) | 0.811 | 0.830 (0.180-3.830) | 0.830 (0.180-3.830) |
| CD117 positive | 0.016 | 0.157 (0.035-0.707) | 0.476 | 0.449 (0.050-4.068) | 0.449 (0.050-4.068) | 0.999 | - | - |
| HLA-DR positive | 0.185 | 3.082 (0.584-16.274) | 0.999 | - | - | 0.999 | - | - |
| Chromosomal karyotype | 0.270 | 2.250 (0.532-9.509) | 0.103 | 3.639 (0.770-17.200) | 3.639 (0.770-17.200) | 0.103 | 3.639 (0.770-17.200) | 3.639 (0.770-17.200) |
| Risk group, high-risk | 0.090 | 2.234 (0.832-12.575) | 0.375 | 2.006 (0.431-9.332) | 2.006 (0.431-9.332) | 0.426 | 0.419 (0.049-3.575) | 0.419 (0.049-3.575) |
| FLT3 mutation | 0.002 | 13.485 (2.679-67.878) | 0.998 | - | - | 0.675 | 0.631 (0.073-5.428) | 0.631 (0.073-5.428) |
| PML/RARα heteromer type, type 3 | 0.061 | 3.893 (0.938-16.152) | 0.052 | 5.245 (0.984-27.958) | 5.245 (0.984-27.958) | 0.613 | 1.486 (0.321-6.882) | 1.486 (0.321-6.882) |
| Fusion rate | 0.782 | 1.005 (0.969-1.042) | 0.114 | 1.057 (0.987-1.133) | 1.057 (0.987-1.133) | 0.315 | 1.027 (0.975-1.081) | 1.027 (0.975-1.081) |
| FAB type, 3v | 0.169 | 5.031 (0.503-50.356) | 0.999 | - | - | 0.999 | - | - |
| BMI (kg/m2) | 0.158 | 1.142 (0.950-1.372) | 0.332 | 1.108 (0.901-1.364) | 1.108 (0.901-1.364) | 0.752 | 0.964 (0.765-1.213) | 0.964 (0.765-1.213) |

**Supplementary Table S5. Multivariable logistic regression model for ED**

| **Unnamed: 0** | **early death events** | **early death events.1** |
| --- | --- | --- |
|  | P-value | HR (95%CI) |
| ECOG PS≥2 | 0.225 | 5.282 (0.358-77.877) |
| PT (s) | 0.857 | 0.958 (0.603-1.522) |
| LDH (U/L) | 0.592 | 1.000 (0.999-1.002) |
| Fibrinogen (g/L) | 0.022 | 0.042 (0.003-0.633) |
| D-dimer (mg/L) | 0.521 | 0.985 (0.940-1.032) |
| Promyelocytic ratio (peripheral blood) | 0.237 | 1.035 (0.978-1.096) |
| CD117 positive | 0.060 | 0.073 (0.005-1.113) |
| FLT3 mutation | 0.047 | 9.934 (1.027-96.100) |

**Supplementary Table S6. Multivariable logistic regression model for relapse**

| **Unnamed: 0** | **disease relapse events** | **disease relapse events.1** |
| --- | --- | --- |
|  | P-value | HR (95%CI) |
| CD34 positive | 0.001 | 44.058 (4.643-418.110) |
| CD56 positive | 0.032 | 9.196 (1.212-69.780) |
